# Patterning Perfluorinated Surface with Graphene Oxide and the Microarray Applications

**DOI:** 10.3390/mi10030173

**Published:** 2019-03-01

**Authors:** Liang Wu, Baishu Liu, Meiling Zhu, Dameng Guo, Han Wu, Liming Bian, Bo Zheng

**Affiliations:** 1 Department of Chemistry, The Chinese University of Hong Kong, Shatin, Hong Kong; juntwl@163.com (L.W.); baishu_liu@163.com (B.L.); guodameng4@gmail.com (D.G.); wuhan_lzu@163.com (H.W.); 2 Department of Biomedical Engineering, The Chinese University of Hong Kong, Shatin, Hong Kong; zhumeil1030@gmail.com (M.Z.); lbian@mae.cuhk.edu.hk (L.B.)

**Keywords:** surface patterning, photolithography, stem cell proliferation, low-level laser therapy, microarray

## Abstract

A method was developed to pattern the surface of perfluorinated materials with graphene oxide thin film, and various biological applications of the patterned perfluorinated surface were illustrated. Perfluorinated surfaces such as Teflon, Cytop, and other perfluorinated materials are known to be both hydrophobic and oleophobic, with low adhesion for most materials. Modifying the perfluorinated surfaces has been difficult due to the extraordinary chemical inertness, which limits the applications of perfluorinated materials as anti-fouling substrates. Herein we successfully patterned Cytop surfaces with graphene oxide. Patterns of the graphene oxide thin film with feature dimension down to 40 microns were formed and remained stable on the Cytop surface against washing with water, ethanol and acetone. The graphene oxide thin film on the Cytop surface allowed non-specific protein adsorption. To illustrate the applications of the patterned Cytop surface, we used the patterned Cytop surface as the substrate to study the protein-protein interactions, stem cell culture, and stem cell proliferation.

## 1. Introduction

Perfluorinated materials are a group of organofluorine compounds in which all the C–H bonds are replaced by C–F bonds. The perfluorinated materials share the well-known properties of extraordinary thermal and chemical stability due to the strong C–F and C–C bonds [1]. Most perfluorinated materials also feature low surface energy and, therefore, display much less non-specific adsorption than other materials. As a result, many perfluorinated materials have been designed and used as anti-fouling coatings for medical and biological applications [2]. On the other hand, it is desirable to modify the perfluorinated surface at predesignated areas for a broader range of applications such as microfluidics [3,4] and microarray technologies [4,5]. The chemical inertness of perfluorinated materials pose substantial difficulty in the surface modification [4]. Methods of modifying perfluorinated materials surface are mainly based on ion beams, laser irradiation, chemical etching, and recently developed polydopamine coating [6,7,8,9]. The high energy ion beams or lasers irradiation require expensive equipment and complicated operation [7,9]. Chemical etching, such as sodium in liquid ammonia [10], sodium naphthalene [11], and FluoroEtch [12], involves highly reactive or corrosive reagents. In addition, both the high energy and chemical treatments increase the surface roughness of perfluorinated materials and are destructive to the surface morphology [8]. Polydopamine coating is a much more gentle surface treatment. In this method, dopamine self-polymerizes into polydopamine thin film on perfluorinated materials surface with high affinity [13]. The as-formed polydopamine has high reactivity towards many different types of function groups, making the coating method useful. However, the coating with polydopamine requires freshly prepared dopamine solution, which increases the experimental complexity. 

To improve the polydopamine coating method, we turned our attention to graphene oxide, which can be stocked in solution for a long time before the coating application. Graphene oxide is the oxidized form of graphene with the oxygen-containing function groups decorating the sp^2^ C basal plane [14]. We hypothesized that graphene oxide would be a promising coating material for perfluorinated surfaces for two reasons. Firstly, the two dimensional feature of graphene oxide would facilitate the non-covalent interaction with the perfluorinated materials. Previous research works have shown that proteins, nucleic acids, and aromatic drug molecules could efficiently adhere to graphene oxide through π-π stacking, electrostatic and hydrophobic interactions [15,16,17]. Graphene oxide surfaces also have good affinity with diverse types of cells, including stem cells [18,19,20,21]. Secondly, the oxygen-containing function groups on graphene oxide would provide the reaction sites for chemical modification. The high chemical activity and tenability of graphene oxide have attracted much attention to use graphene oxide in the biosensor designs [14,22,23]. Based on the hypothesis in the current work we aimed to test graphene oxide as a new coating material for perfluorinated surfaces and to further apply the graphene oxide patterned perfluorinated surfaces to the fabrication of protein and cell microarrays. 

## 2. Materials and Methods 

### 2.1. Materials

Cytop^TM^ (CTL-809M) and the fluorinated solvent CT-Solv.180 were purchased from Asashi Glass Co (Tokyo, Japan). Graphite was from International Laboratory, USA (South San Francisco, CA, USA). AZ P4620 photoresist was from MicroChemicals GmbH (Ulm, Germany). Sodium nitrate and hydrogen peroxide were from Scharlab S. L (Barcelona, Spain). Sulfuric acid (98%) was from RCI Labscan Limited (Bangkok, Thailand). Hydrochloric acid was from Fisher Scientific (Waltham, MA, USA). Potassium permanganate, ZONYL FSO-100 fluorosurfactant, trichloro (1H, 1H, 2H, 2H-perfluorooctyl) silane (97%) were from Sigma-Aldrich (St. Louis, MO, USA). β-human chorionic gonadotrophin (β-hCG) and fluorescein isothiocyanate (FITC) labeled anti-β-hCG were from Abcam (Cambridge, UK). Mesenchymal stem cells were from mouse bone marrow (Astarte Biologics, Inc., Bothell, WA, USA). Dulbecco’s phosphate buffered saline (DPBS 1X), minimum essential medium (MEM) alpha (1X), and trypsin were from Gibco (Thermo Fisher Scientific, Waltham, MA, USA).

### 2.2. Synthesis of Graphene Oxide

Graphene oxide was synthesized using the modified Hummer’s method [24]. Five grams of graphite fine powder, 2.5 g sodium nitrate, and 120 mL sulfuric acid (98%) were mixed and stirred for 30 min in the room temperature. Fifteen grams of potassium permanganate powder were slowly added into the mixture under the protection of ice bath. The adding rate was limited by controlling the temperature of the reaction system below 20 °C. Afterwards, the reaction system was transferred to the water bath at 30 °C and stirred overnight. A total of 150 mL distilled water was then slowly added to the reaction system followed by stirring for one day. Afterwards, 30% hydrogen peroxide was added to the reaction system followed by stirring overnight. The reaction mixture was then filtered using vacuum filtration method and washed with 1 L 5% hydrochloric acid. The acquired precipitant was rinsed with distilled water until the pH of filtrate became 7. The precipitants were then dissolved in distilled water and treated with ultrasound for 30 min. After still stratification overnight, the supernatant liquor was collected as the graphene oxide solution.

### 2.3. Patterning Cytop Surface with Graphene Oxide Thin Film

Glass slides were cleaned with piranha solution, a mixture of 98% sulfuric acid and 30% hydrogen peroxide with 3:1 volume ratio, and were dried in the oven. The glass slides were then spin coated with 1% Cytop solution (1% in CT-Solv.180) at a 500 rpm for 30 s and heated at 180 °C for 1 h. After cooling down naturally, Cytop formed a uniform layer on the glass slide. The Cytop surface was then spin coated with AZ photoresist (AZ P4620, MicroChemicals, Ulm, Germany) at a speed of 2000 rpm for 30 s. The coated AZ photoresist was heated in the 100 ℃ heater for 5 min. Then a photomask containing two types of surface, transparent to pass the light and black to block the light, was placed onto the AZ photoresist. UV irradiation at 365 nm above the photomask was turned on for 30 s. A 0.02 M NaOH solution was used to wash the AZ photoresist. The area on the AZ photoresist irradiated with UV light became soluble in the NaOH solution while the non-irradiated area was insoluble and remained on the substrate, and AZ patterns corresponding to the photomask were created on the Cytop surface. A graphene oxide solution with 0.025% ZONYL FSO-100 fluorosurfactant was added onto the AZ-patterned Cytop surface to form graphene oxide thin films. Afterwards, the graphene oxide-patterned Cytop surface was placed in a vacuum desiccator which contained saturated vapor of trichloro(1H,1H,2H,2H-perfluorooctyl)silane. After the fluorosilane treatment, the graphene oxide patterned Cytop surface was then treated by oxygen plasma (SPI Plasma Prep II) for 2 min. In the final step, acetone developing or peeling using adhesive tape was performed to remove the AZ photoresist from the Cytop surface. 

### 2.4. Surface Testing and Contact Angle Measurement

Aqueous solution of rhodamine with the concentration of 0.50 mg/mL was prepared to test the surface property of the graphene oxide patterned Cytop surface. A large drop of rhodamine solution was placed on top of the patterned Cytop surface, followed by tilting the surface so that the drop of rhodamine solution flowed off the surface.

Three Cytop surfaces presenting the graphene oxide patterns, the fluorosilane treated graphene oxide patterns, the fluorosilane and plasma treated graphene oxide patterns, were placed onto a flat surface of the optical tensiometer (CA-XP, Kyowa Interface Science, Niiza, Japan). The optical tensiometer produced a water droplet onto each surface. A camera then took a photo of the water droplet resting on each surface. By manually selecting the highest point of the water droplet and two contacting points of water/air/solid interfaces, the tensiometer calculated the water contact angles.

### 2.5. Scanning Electron Microscopy (SEM)

The graphene oxide solution was deposited onto a silicon substrate, which was then treated by freeze drying to remove the solvent and form the graphene oxide thin film. The silicon substrate was then loaded into a thermal evaporator and coated by a thin layer of Au. Finally, the silicon substrate was loaded into the scanning electron microscopy (SEM) to observe the micro structures of the graphene oxide.

### 2.6. Confocal Microscopy

The stem cells microarray supported by the graphene oxide patterned Cytop substrate was put onto the holder of the confocal microscope (C1, Nikon, Tokyo, Japan). A 488 nm excitation laser was used to obtain the fluorescence image.

### 2.7. X-ray Diffraction (XRD), X-ray Photoelectron Spectroscopy (XPS), and Infrared (IR) Spectroscopy

Graphite and graphene oxide samples were ground into fine powders and then casted onto the glass slides. The casted powders were then pressed to form flat sample surface for X-ray diffraction (XRD) measurement. The samples were scanned from 5–40 degrees in XRD. 

To prepare the samples for XPS characterization, the graphene oxide solution with 0.025% ZONYL FSO-100 fluorosurfactant was deposited on the Cytop coated glass slide to form graphene oxide thin films. The thin film was treated with saturated vapor of trichloro(1H,1H,2H,2H-perfluorooctyl)silane in a vacuum desiccator for the fluorosilane treatment. In the final step, the graphene oxide thin film was peeled off by conductive tape and loaded into the X-ray photoelectron spectroscopy (XPS) spectrometer (VG Escalab 220i-XL, AlKα X-ray) for XPS characterization. 

For infrared (IR) spectroscopy, graphite and graphene oxide samples were ground into fine powders and dried in a 70 ℃ oven. Two-gundred milligram KBr and 2 mg graphite or graphene oxide powders were mixed together and ground in one direction. The ground powders were put into the mold and pressed to become the sample tablet, which was used in the IR spectrometer for IR testing.

### 2.8. Stem Cells Culture and Harvest

Mesenchymal stem cells (MSCs) derived from green fluorescent protein (GFP) transgenic mice were used for the low-level laser irradiation study. The MSCs were taken out from liquid nitrogen and quickly warmed up at 37 ℃ water bath. Then the MSCs were added into a 15 mL centrifugal tube containing 5 mL growth media. The tube was centrifuged at 500× *g* for 3 min to remove the liquid. Five milliliters of new growth media was added into the tube to suspend the MSCs. Afterwards, the MSCs suspension was uniformly added into 100 mm petri dish containing 5 mL new growth media. The petri dish was put into a 37 ℃ incubator for the MSCs proliferation. After two days, the petri dish was taken out from the incubator and observed under microscope. A 70% to 80% coverage ratio of the MSCs would represent nearly one million cells inside the petri dish. The growth media inside the petri dish was removed by pipetting, followed by rinsing with 5 mL phosphate-buffered saline (PBS) buffer twice. Two milliliters of trypsin solution was added into the petri dish. The petri dish was then put into 37 ℃ incubator for 5 min, followed by the addition of 4 mL growth media. Pipette was used to blow the wall of the petri dish to completely detach the MSCs. The solution containing the MSCs inside the petri dish was transferred into the centrifugal tube and underwent centrifugation at 500× *g* for 3 min. 0.5 mL new growth media was added into the tube after the supernatant was removed. The collected stem cells were mixed with the newly added growth media. Fifteen microliters of mixed solution was taken out and mixed with 15 μL trypan blue, followed by injection into the hemocytometer. A total of 0.78 million MSCs were harvested as a result. 

### 2.9. Culturing Stem Sells on Graphene Oxide and Irradiation with a Low-Level Laser

**Figure 1:**
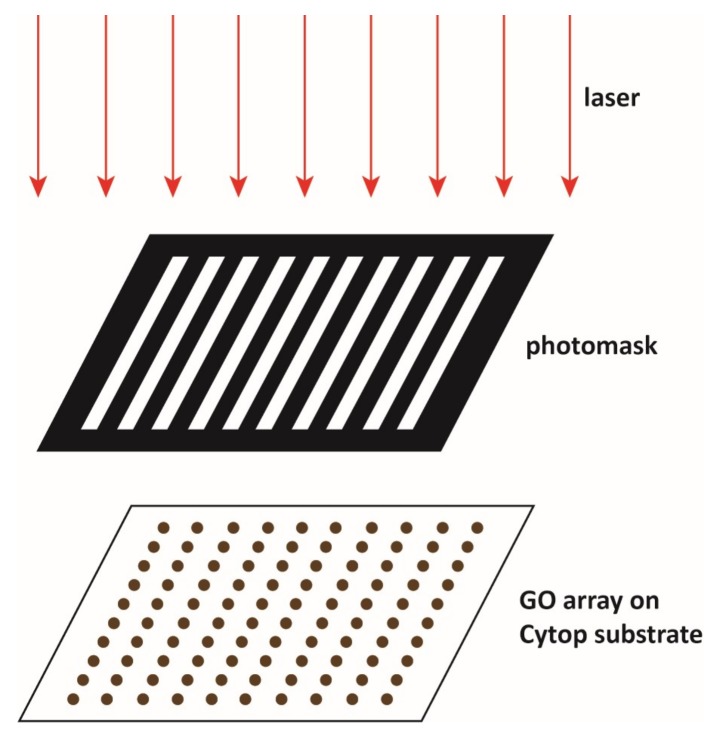
Setup of the gradient of low-level laser irradiation on the cultured stem cells.

The graphene oxide patterned Cytop surface was sterilized under ultraviolet (UV) irradiation in the fume hood. A total of 0.2 million MSCs from the 0.78 harvested MSCs were uniformly distributed onto this sterilized surface. After one day’s culture in the 37 ℃ incubator, the Cytop surface was washed with PBS buffer for three times to remove the MSCs on the unmodified Cytop surface. The MSCs remained only on the graphene oxide coated area. The remaining stem cells were then irradiated for 10 min using the low-level 633 nm laser (Uniphase Novette 1507-0, 1.1 mW, Figure 1) through a photomask. As shown in Figure 1, the photomask contains 10 grey lines with increasing darkness, which allowed a certain transmission percentage of the incoming laser irradiation from 0% to 100% with 11.11% step increase. After the laser irradiation, the MSCs on the patterned Cytop surface were put back into the 37 ℃ incubator. The MSCs proliferated for 24 hours in the incubator. After 24 hours proliferation, the petri dish was taken out and the growth media was pipetted out. PBS buffer was used to wash the Cytop surface for three times, and the MSCs were examined under the confocal microscope. The proliferation ratio was calculated as the ratio of the fluorescence from the stem cells after irradiation and culture to the fluorescence before the irradiation.

## 3. Results and Discussion

**Figure 2:**
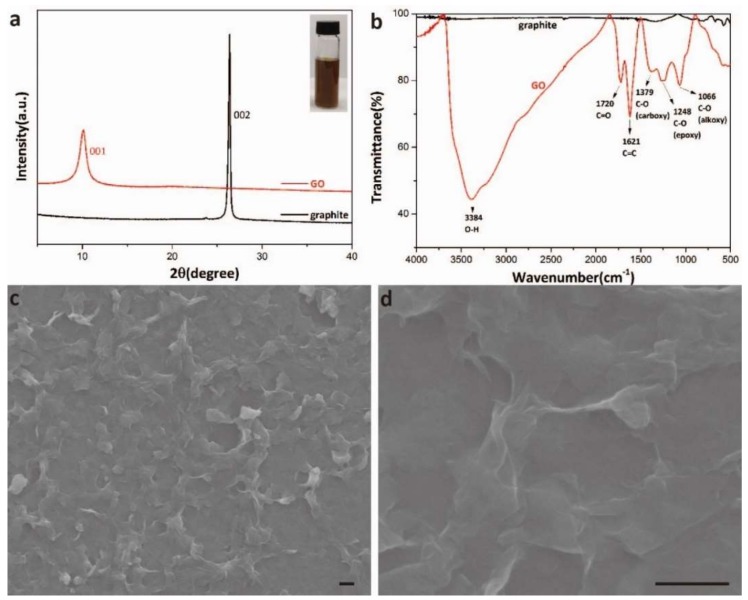
Characterization of the synthesized graphene oxide; (**a**) X-ray diffraction (XRD) image of the graphite (black) and graphene oxide (red). The inset is the optical image of the graphene oxide solution; (**b**) infrared (IR) analysis of the graphite (black) and graphene oxide (red); and (**c**,**d**) scanning electron microscopy (SEM) images of the graphene oxide. Scale bars: 1 μm.

The IR spectroscopy, XRD analysis and SEM characterization of the graphene oxide were performed to confirm the synthesis result (Figure 2). The typical diffraction peak of graphene oxide at about 10 degrees was clearly seen after the synthesis (Figure 2a) [25]. The inset image in Figure 2a showed the typical brown color of graphene oxide solution. The oxygen-containing functional groups were characterized by IR (Figure 2b). The O–H group at 3384 cm^−1^, C-O groups at 1379 cm^−1^, 1248 cm^−1^, and 1066 cm^−1^, and the C=O group at 1720 cm^−1^ indicated that a large number of oxygen-containing functional groups appeared on graphene oxide surface. The nanosheet structure of graphene oxide can be observed from SEM images (Figure 2c,d), which presented nanoscale surface roughness. The IR, XRD analysis, and SEM characterization demonstrated the successful synthesis of graphene oxide. 

**Figure 3:**
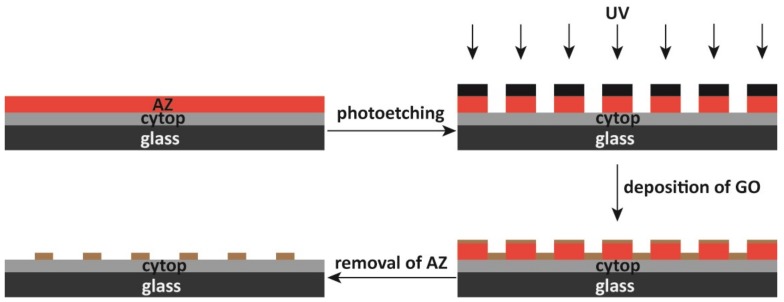
Scheme of patterning Cytop surface by graphene oxide using photolithography.

**Figure 4:**
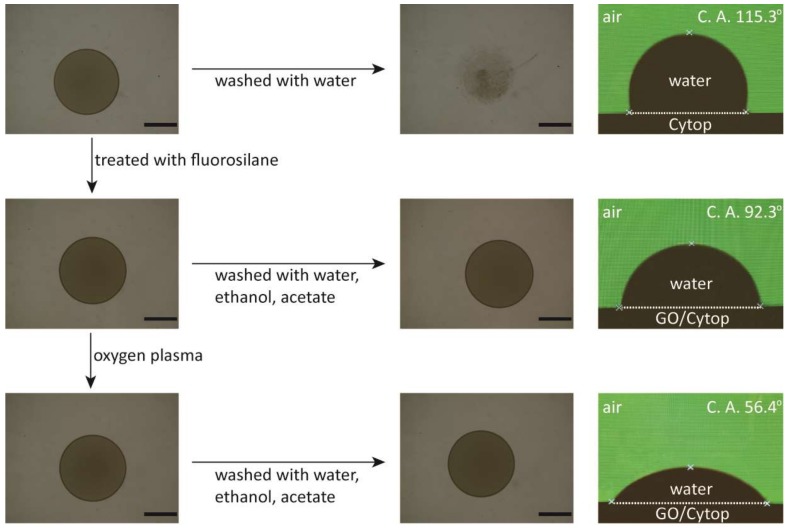
Left and center columns: Optical images of the graphene oxide thin films on Cytop with fluorosilane treatment and then oxygen plasma treatment. Right column: The images from the optical tensiometer for the contact angle measurements. Scale bars: 5 mm.

We used photolithography to form different sizes and shapes of graphene oxide patterns on Cytop surfaces (Figure 3). During the photolithography process, we observed that the addition of ZONYL FSO-100 fluorosurfactant in the graphene oxide solution promoted the adhesion to the perfluorinated material surface [8]. However, after the drying of the graphene oxide solution to form the graphene oxide thin film, we found that water could still wash away the graphene oxide thin film (Figure 4). To solve this problem, we found that the treatment of fluorosilane was able to stabilize the graphene oxide thin film against washing by water, ethanol and acetone. The XPS result of the graphene oxide thin film showed no detectable presence of Si atoms on the film’s backside, i.e., the side facing the Cytop surface (Figure S1). Our hypothesis is that the fluorosilane treatment led to the fluorosilane condensation along the edge of the graphene oxide film [26]. The condensation product would help maintain ZONYL FSO-100 fluorosurfactant molecules in between the graphene oxide film and Cytop surface, which stabilized the graphene oxide coating against solvent washing through the fluorous-fluorous and solvophobic interactions [27,28,29]. Oxygen plasma treatment turned the outer surface of the graphene oxide thin film hydrophilic again (Figure 4). The final step of the removal of the AZ photoresist (Figure 3) produced different graphene oxide patterns on the Cytop surfaces. 

**Figure 5:**
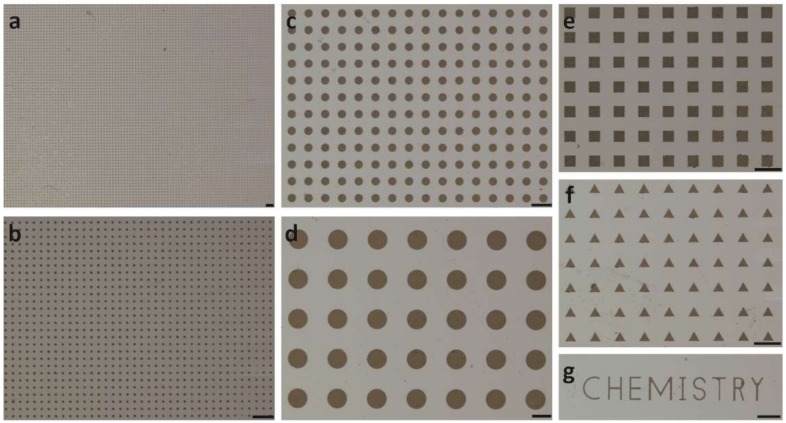
Optical images of the multiple graphene oxide patterns modified on Cytop surface. (**a**) A 100 × 100 array of graphene oxide circles with 50 μm diameter. (**b**) The blow-up of part of (**a**). (**c**) An array of graphene oxide circles with 200 μm diameter. (**d**) An array of graphene oxide circles with 500 μm diameter. (**e**) An array of graphene oxide squares with 200 μm side length. (**f**) An array of graphene oxide triangles with 200 μm side length. (**g**) “CHEMISTRY” letters made of graphene oxide thin film. Scale bars: 500 μm.

Using AZ photoresist as a protecting layer, graphene oxide thin film with different sizes and shapes including circles, squares, triangles, and complex letters could be patterned on Cytop surface (Figure 4). Graphene oxide circles arrays with diameters ranging from 50 μm to 500 μm were formed on Cytop surfaces (Figure 5a–d). The 100 × 100 arrays of graphene oxide circles, squares, and triangles with sharp edge and vertex were easily formedon Cytop surface (Figure 5e,f). More complex features such as letters were also produced on the Cytop surface (Figure 5g). 

**Figure 6:**
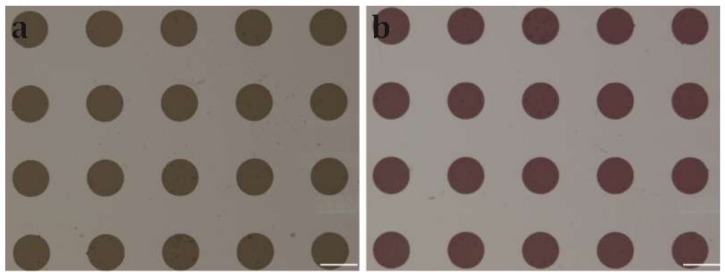
Optical images of the hydrophilic graphene oxide thin film array modified on hydrophobic Cytop surface. (**a**) The graphene oxide thin film array at dry state. (**b**) The graphene oxide thin film array with droplets of rhodamine solution pinned on top of the graphene oxide. Scale bars: 500 μm.

The patterned Cytop surface facilitated simple and rapid reagent loading due to the hydrophobicity contrast between Cytop and graphene oxide. For example, we flowed the aqueous Rhodamine solution over the patterned Cytop surface (Figure 6a), and we observed that the rhodamine solution was pinned on only graphene oxide thin film while no rhodamine solution remained on the Cytop surface (Figure 6b). 

**Figure 7:**
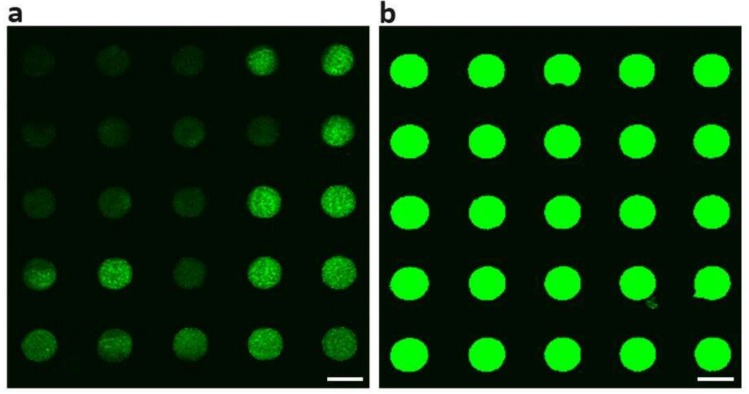
Confocal microscope images showing the non-specific protein adsorption on graphene oxide thin film array. (**a**) Fluorescein isothiocyanate (FITC)-labeled anti-β-hCG adsorbed on graphene oxide thin film array. (**b**) β-hCG adsorbed on graphene oxide thin film array, followed by incubation with FITC-labeled anti-β-hCG. Scale bars: 200 μm.

The patterned Cytop surface facilitated the fabrication of protein microarrays due to the non-specific adsorption of proteins on graphene oxide and the anti-fouling property of perfluorinated surfaces. We investigated the protein adsorption on the graphene oxide modified Cytop surface (Figure 7). Fluorescein isothiocyanate (FITC)-labeled anti-β-human chorionic gonadotropin (anti-β-hCG) solution was incubated with the entire graphene oxide-modified Cytop surface overnight at 4 °C. After washing the entire platform with PBS buffer, we found that the FITC-labeled anti-β-hCG only adsorbed on the graphene oxide thin film array while there was no observable adsorption on Cytop surface (Figure 7a). This observation demonstrated the successful selective modification of the chemically inert and hydrophobic Cytop surface with a chemically active and hydrophilic graphene oxide thin film array. This result was also consistent with the previous reports of immobilizing protein molecules on graphene oxide, which was based on the non-covalent interactions between the protein molecules and graphene oxide, e.g., electrostatic interactions and hydrogen bonding [30,31]. Meanwhile, only weak green fluorescence was observed in the graphene oxide thin film array, which was due to the quenching effect of graphene oxide [32]. To avoid the quenching effect, we first treated the entire graphene oxide modified Cytop surface with β-hCG solution, and then incubated the substrate with FITC labeled anti-β-hCG. The adsorbed β-hCG on graphene oxide greatly increased the distance between graphene oxide and FITC-labeled anti-β-hCG. The quenching effect of graphene oxide greatly decreased. A strong green fluorescence array of FITC-labeled anti-β-hCG was observed on the Cytop substrate (Figure 7b). The simple and reliable protein immobilization on graphene oxide makes this platform useful in the construction of protein or peptide microarrays, which are an important tool in high-throughput bioanalysis. 

**Figure 8:**
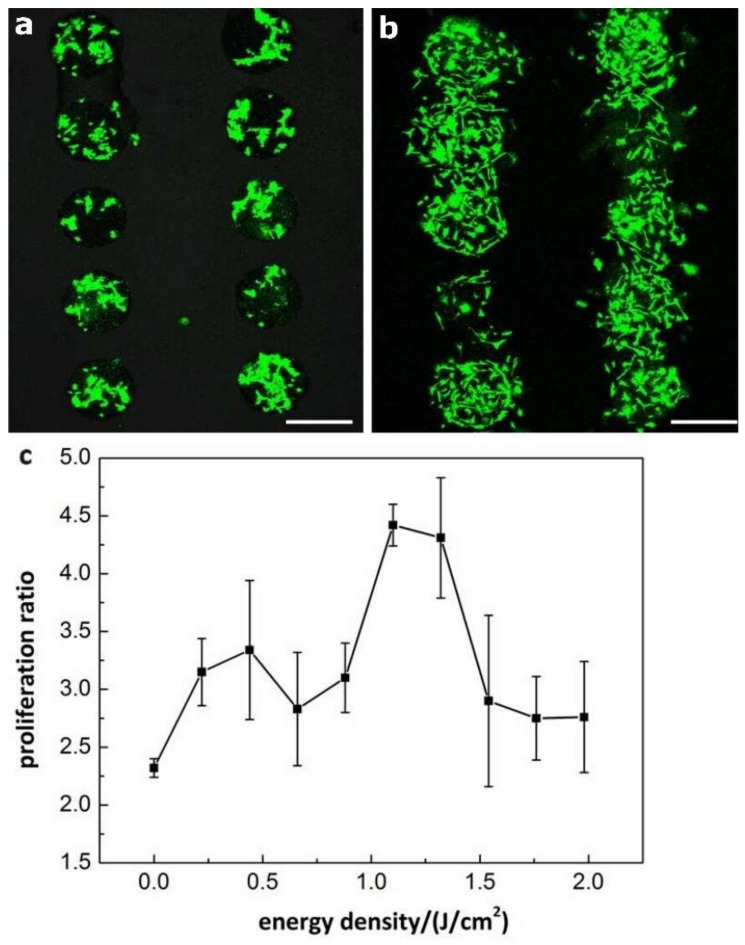
The proliferation condition of the green fluorescent protein (GFP) labeled mouse mesenchymal stem cells (MSC) on array of graphene oxide thin film on Cytop. (**a**) Confocal microscope image of the stem cells adhering to and growing on only graphene oxide thin film after washing with phosphate-buffered saline (PBS) buffer. (**b**) Confocal microscope image of the stem cells after irradiation with low-level laser light and culture for 24 h. (**c**) The relationship between the stem cell proliferation ratio and the energy density of the low-level laser light. The proliferation ratio was the average of three parallel measurements. The confocal microscope images for each irradiation energies are available as Figures S2–S11 in Supplementary Materials. Scale bars: 500 μm.

The patterned Cytop surface also facilitated the fabrication of cell microarrays. Previously, researchers demonstrated that low-level laser irradiation could stimulate the proliferation of many different types of cultured cells, including stem cells [33]. For example, laser irradiation at 635 nm and 0.5 J/cm^2^ significantly stimulated the proliferation of bone marrow derived MSCs from rats and mice [34,35]. The laser energy density seemed a critical factor to stimulate the MSCs. Lasers with an optimally low energy density could significantly enhance the proliferation of stem cells, while a high energy density could lead to limited enhancement or even inhibition effects [36]. Despite the many reports of the enhancement effect of the low-level laser irradiation on the MSCs proliferation, the molecular mechanism is not fully understood. A convenient method of producing the MSCs microarray for the research of the low-level laser irradiation is therefore highly desirable. The anti-fouling Cytop surface patterned with the cytocompatible graphene oxide provided a promising substrate for the MSCs microarrays [37]. Graphene oxide substrate has been suggested to promote the growth and proliferation of certain types of stem cells [38,39] but bring no obvious changes in the growth and proliferation for some other types of stem cells [40,41]. In the current work we observed that the GFP labeled mouse MSCs adhered to only the graphene oxide thin film array but not the Cytop surface after washing with PBS buffer (Figure 8a) [42]. The proliferation condition of the stem cells was studied using the stem cell microarray formed on the Cytop surface patterned with the array of graphene oxide. After the irradiation by the low-level laser irradiation (633 nm, 1.1 mW) and culture for 24 h, the stem cells proliferated (Figure 8b). Using the graphene oxide thin film array, the relationship between the stem cell proliferation rate and the energy density of the low-level laser light was investigated. The photomask allowed us to produce a gradient of the laser irradiation density across the stem cell microarray (Figure 1). As a result, we demonstrated the screening with a single laser source and culture of the stem cells (Figure 8c). We could find that the low-level laser light indeed promoted the proliferation rate of the stem cells, comparing the result of the control with no laser irradiation. We concluded that the energy density in the range from 1.10 to 1.32 J/cm^2^ of the low-level laser light (633 nm) was optimal to promote the proliferation of the mouse mesenchymal stem cells. 

## 4. Conclusions

In conclusion, we have developed a new gentle method of modifying perfluorinated surfaces with graphene oxide thin film. The chemically inert and hydrophobic perfluorinated material surface can be patterned through photolithography with functionalized and hydrophilic graphene oxide thin film. The graphene oxide thin film on the perfluorinated material surface was stable against washing with water, ethanol, and acetone. Owing to the high adsorption capacity for serum proteins, the patterned graphene oxide presented the potential to be an excellent substrate for the stem cells culture. We applied this patterned perfluorinated surface to screening the conditions of the stem cells’ proliferation under the low-level laser irradiation. The graphene oxide-patterned perfluorinated materials could potentially be useful as a substrate for microarray fabrication in applications, such as high throughput bioanalysis.

## Supplementary Materials

The following are available online at https://www.mdpi.com/2072-666X/10/3/173/s1, Figure S1: XPS spectra of the graphene oxide film; Figure S2: Confocal microscope image of the stem cells on graphene oxide thin film before irradiation with energy densities of 1.98 J/cm^2^ and 1.76 J/cm^2^; Figure S3: Confocal microscope image of the stem cells on graphene oxide thin film before irradiation with energy densities of 1.54 J/cm^2^ and 1.32 J/cm^2^; Figure S4: Confocal microscope image of the stem cells on graphene oxide thin film before irradiation with the energy density of 1.10 J/cm^2^ and 0.88 J/cm^2^; Figure S5: Confocal microscope image of the stem cells on graphene oxide thin film before irradiation with energy densities of 0.66 J/cm^2^ and 0.44 J/cm^2^; Figure S6: Confocal microscope image of the stem cells on graphene oxide thin film before irradiation with the energy density of 0.22 J/cm^2^ and 0 J/cm^2^; Figure S7: Confocal microscope image of the stem cells on graphene oxide thin film after irradiation with energy densities of 1.98 J/cm^2^ and 1.76 J/cm^2^; Figure S8: Confocal microscope image of the stem cells on graphene oxide thin film after irradiation with energy densities of 1.54 J/cm^2^ and 1.32 J/cm^2^; Figure S9: Confocal microscope image of the stem cells on graphene oxide thin film after irradiation with energy densities of 1.11 J/cm^2^ and 0.98 J/cm^2^; Figure S10: Confocal microscope image of the stem cells on graphene oxide thin film after irradiation with energy densities of 0.66 J/cm^2^ and 0.44 J/cm^2^; Figure S11: Confocal microscope image of the stem cells on graphene oxide thin film after irradiation with energy densities of 0.22 J/cm^2^ and 0 J/cm^2^.
