# Supplementary material for: Patterning Perfluorinated Surface with Graphene Oxide and the Microarray Applications
Source: Micromachines (Basel). 2019 Mar 1;10(3):173. doi: 10.3390/mi10030173 (PMC6470711; doi:10.3390/mi10030173)
Supplement: Supplementary file 1 [file micromachines-10-00173-s001.pdf]

Supplementary Materials: Patterning Perfluorinated Surface with Graphene Oxide and the Microarray Applications

Liang Wu, Baishu Liu, Meiling Zhu, Dameng Guo, Han Wu, Liming Bian, and Bo Zheng

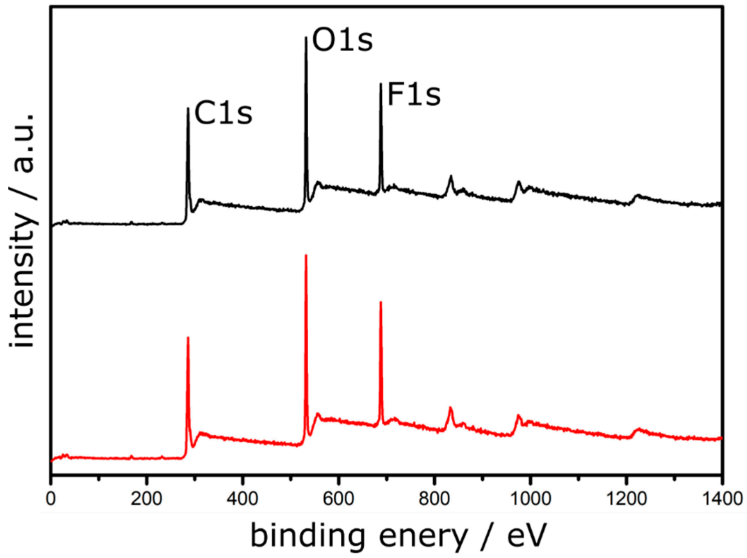

Figure S1. X-ray photoelectron spectroscopy (XPS) spectra of the graphene oxide film before (black) and after (red) the treatment by trichloro (1H, 1H, 2H, 2H-perfluorooctyl) silane.

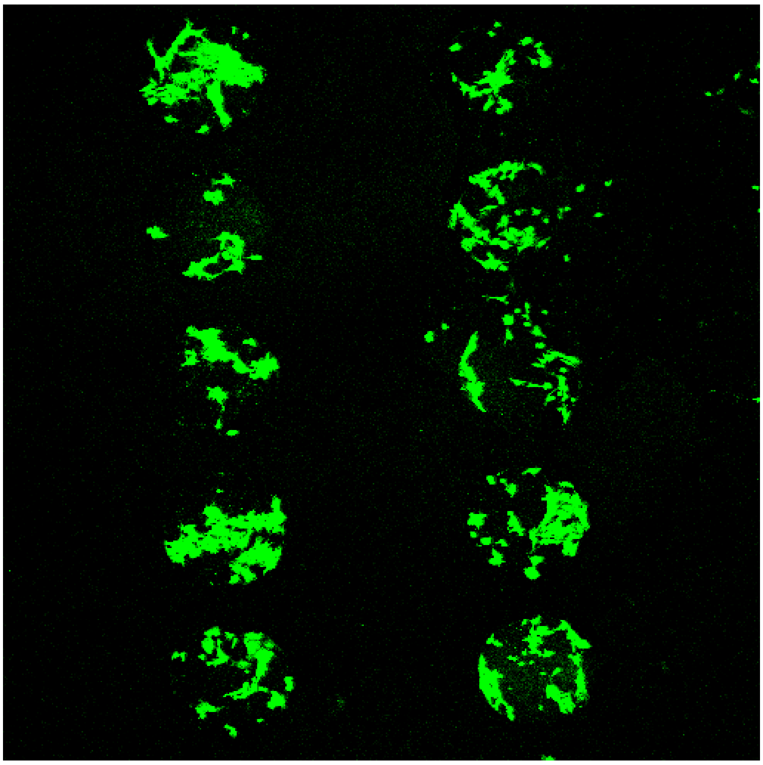

Figure S2. Confocal microscope image of the stem cells, adhering to and growing on only a graphene oxide thin film before irradiation with the energy density of 1.98 J/cm^2 (right column) and 1.76 J/cm^2 (left column)

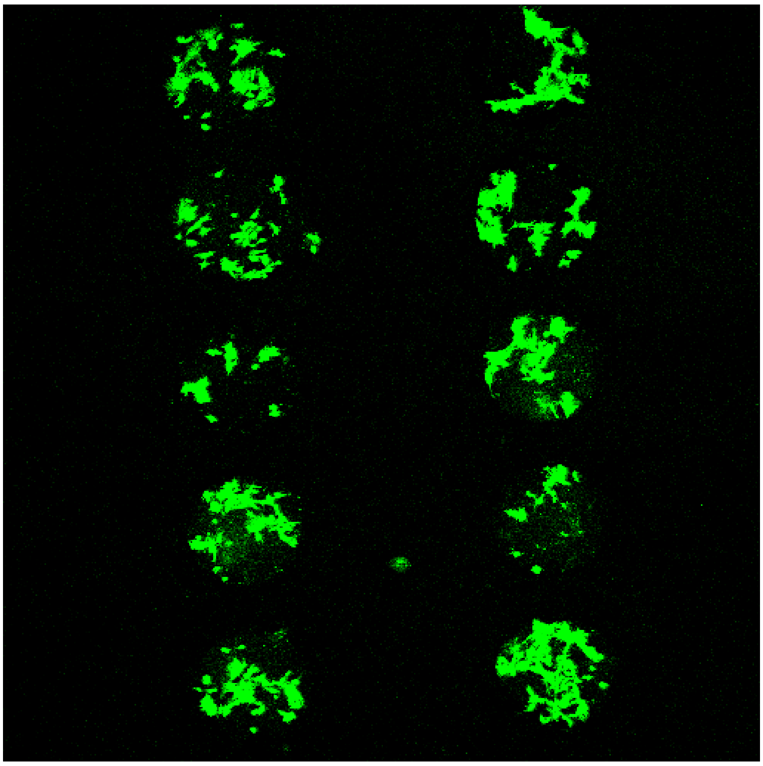

Figure S3. Confocal microscope image of the stem cells, adhering to and growing on only a graphene oxide thin film before irradiation with the energy density of 1.54 J/cm^2 (right column) and 1.32 J/cm^2 (left column).

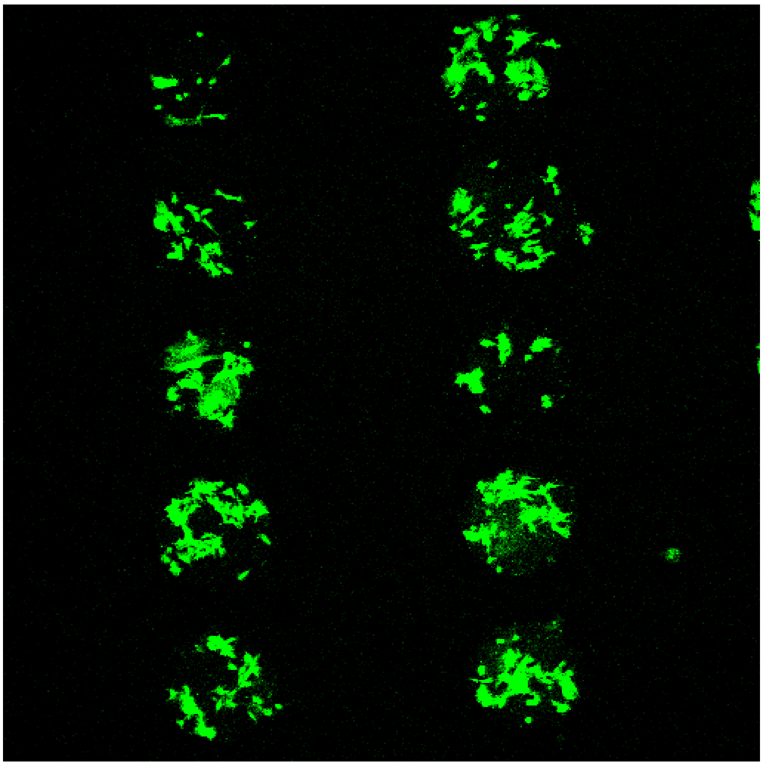

Figure S4. Confocal microscope image of the stem cells, adhering to and growing on only a graphene oxide thin film before irradiation with the energy density of 1.10 J/cm^2 (right column) and 0.88 J/cm^2 (left column).

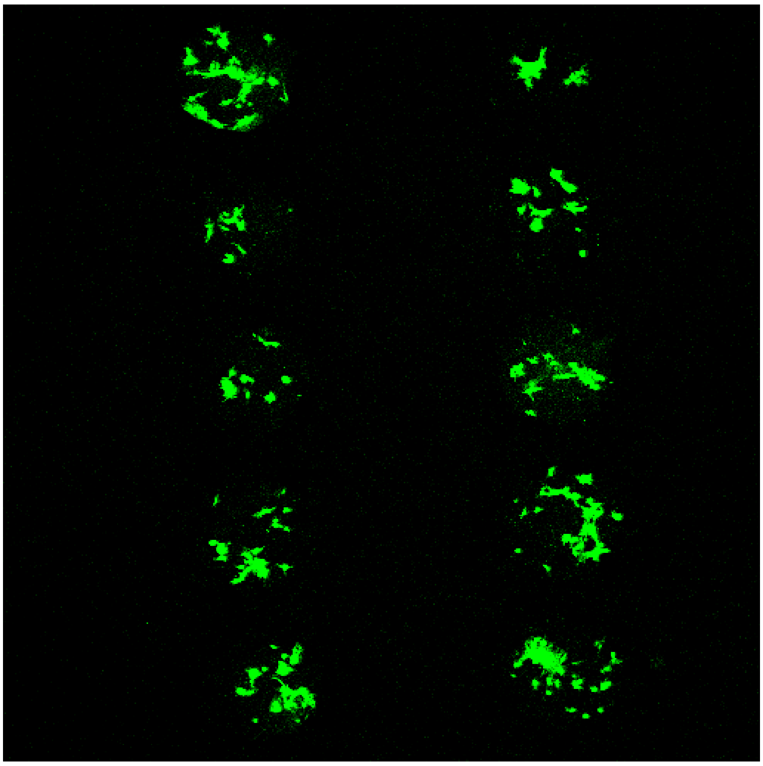

Figure S5. Confocal microscope image of the stem cells, adhering to and growing on only a graphene oxide thin film before irradiation with the energy density of 0.66 J/cm^2 (right column) and 0.44 J/cm^2 (left column).

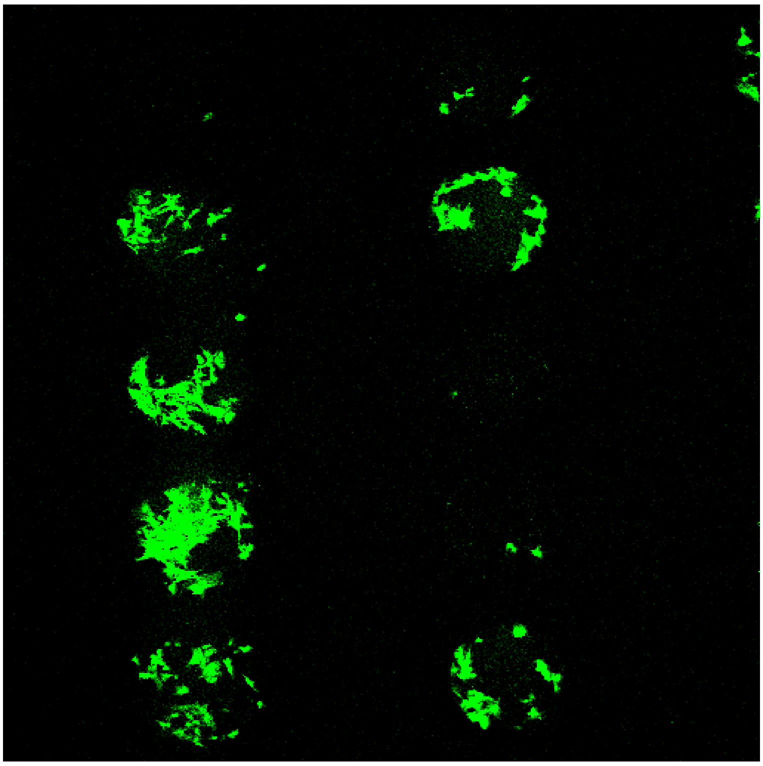

Figure S6. Confocal microscope image of the stem cells, adhering to and growing on only a graphene oxide thin film before irradiation with the energy density of 0.22 J/cm^2 (right column) and 0 J/cm^2 (left column).

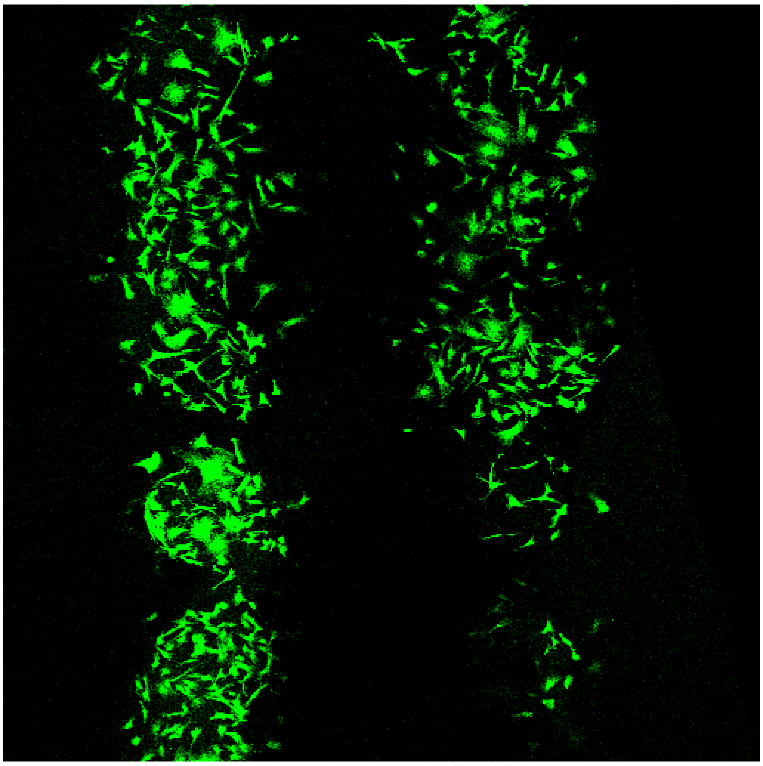

Figure S7. Confocal microscope image of the stem cells, adhering to and growing on only a graphene oxide thin film after irradiation and culture. The right column correlates to the energy density of 1.98 J/cm^2. The left column correlates to the energy density of 1.76 J/cm^2.

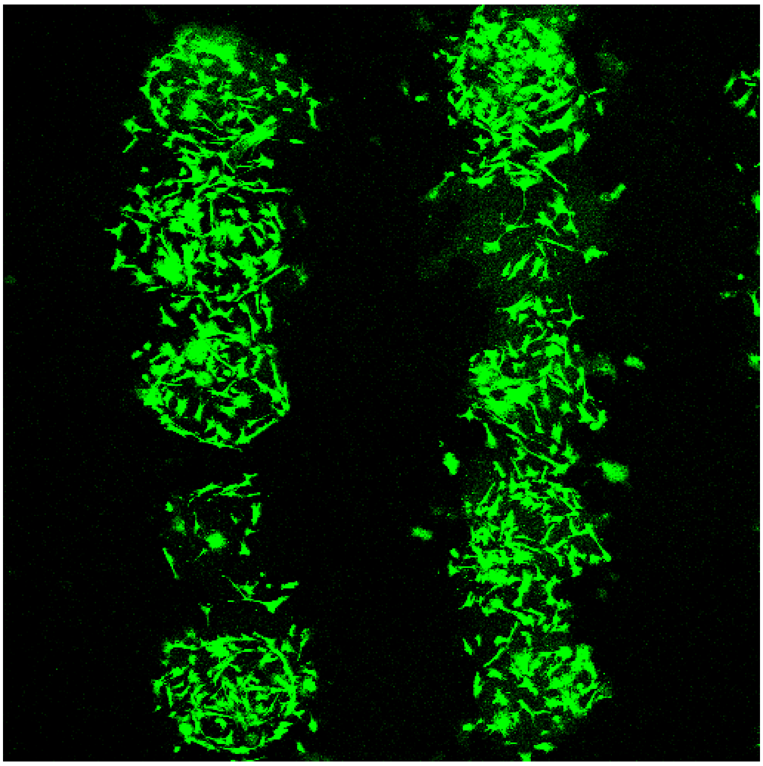

Figure S8. Confocal microscope image of the stem cells, adhering to and growing on only a graphene oxide thin film after irradiation and culture. The right column correlates to the energy density of 1.54 J/cm^2. The left column correlates to the energy density of 1.32 J/cm^2.

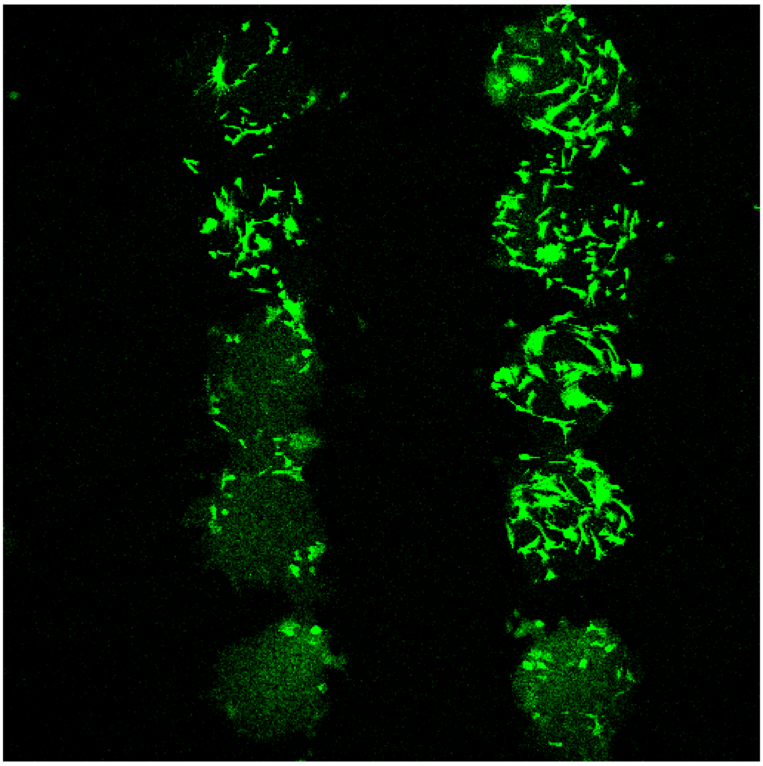

Figure S9. Confocal microscope image of the stem cells, adhering to and growing on only a graphene oxide thin film after irradiation and culture. The right column correlates to the energy density of 1.11 J/cm^2. The left column correlates to the energy density of 0.98 J/cm^2.

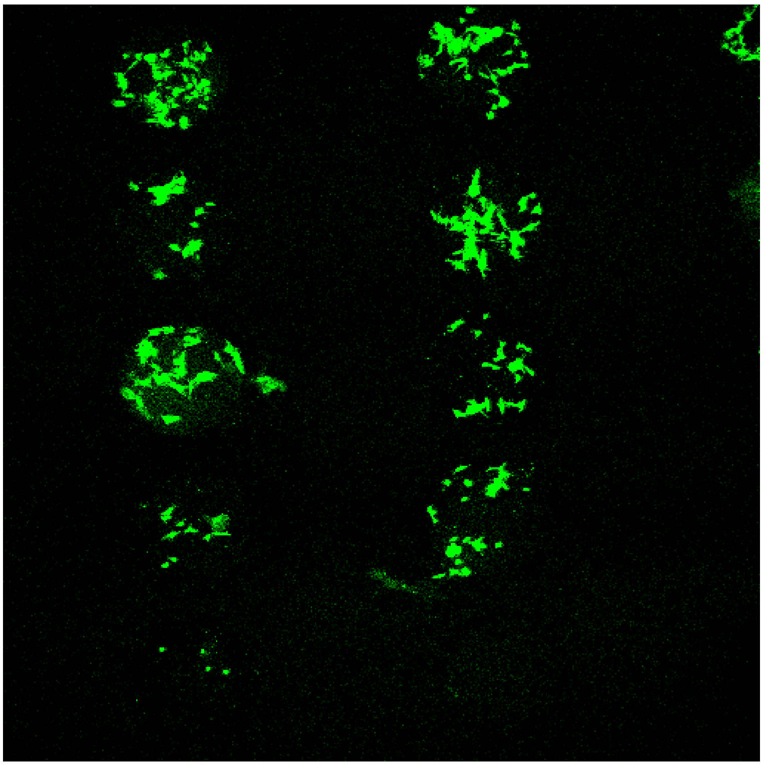

Figure S10. Confocal microscope image of the stem cells, adhering to, and growing on only a graphene oxide thin film after irradiation and culture. The right column correlates to the energy density of 0.66 J/cm^2. The left column correlates to the energy density of 0.44 J/cm^2.

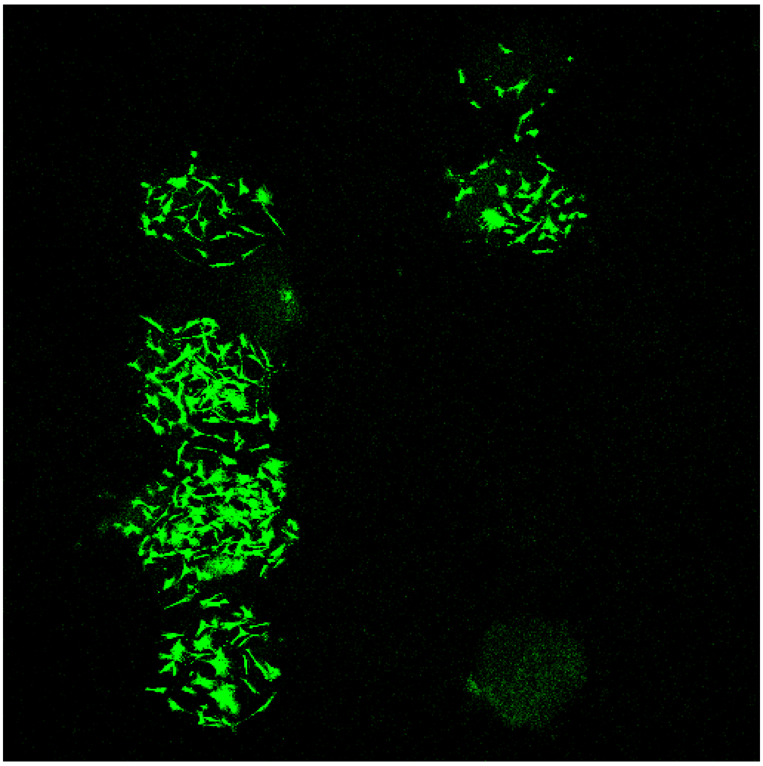

Figure S11. Confocal microscope image of the stem cells, adhering to and growing on only graphene oxide thin film after irradiation and culture. The right column correlates to the energy density of 0.22 J/cm^2. The left column correlates to the energy density of 0 J/cm^2.
